# Hemichorea-Hemiballismus Syndrome in Acute Non-ketotic Hyperglycemia

**DOI:** 10.7759/cureus.19026

**Published:** 2021-10-25

**Authors:** Amr Salem, Abdelilah Lahmar

**Affiliations:** 1 Hospital Medicine, Boston University School of Medicine, Boston, USA; 2 Medicine, University Mohammed VI University Hospital, Faculty of medicine and pharmacy in Oujda, Oujda, MAR

**Keywords:** hyperglycemia, hemichorea, hemiballismus, diabetes, acute care

## Abstract

Hemichorea/hemiballismus syndrome secondary to non-ketotic hyperglycemia is a movement disorder induced by long-standing poor control of diabetes mellitus. Diagnosis is based on clinical assessment and imaging. Here we report a rare case of a 56-year-old woman presenting with involuntary movements on the left side secondary to acute hyperglycemia. She received antidiabetic and anti-choreic drugs. The patient's glycemic profile was closely monitored, and she, consequently, responded favorably to therapy.

## Introduction

Hemichorea/hemiballismus syndrome associated with non-ketotic hyperglycemia is an uncommon initial presentation of diabetes mellitus or occurrence in diabetic patients with poor glycemic control [1]. This syndrome is usually unilateral but can rarely present bilaterally. It is sometimes referred to as “diabetic striatopathy” since it is consistent with striatal hyperintensity on T1-weighted magnetic resonance imaging (MRI) in diabetic patients exhibiting a contralateral movement disorder [2]. Hemichorea/hemiballismus has mainly been described in elderly female patients of Asian ethnicity, representing 71.6% of reported cases. However, an increasing number of cases have been reported in Europe, North America, and Latin America. The condition is potentially underdiagnosed in the western population [3, 4]. The pathophysiology is unclear, although several hypotheses have been proposed. Treatment consists of managing acute hyperglycemia, and resolution of the syndrome takes between two and 28 days. It may, however, persist in some cases [5].

To the best of our knowledge, there has only been one study of four patients from Morocco with this syndrome [6]. Here we report a case report of a female patient presenting with hemichorea/hemiballismus syndrome and non-ketotic hyperglycemia.

## Case presentation

A 56-year-old Moroccan woman without any previous medical history presented with a two-week history of involuntary movements affecting the left upper and lower limbs.

On presentation, she had discontinuous focal dystonia of her left hand. However, over the next few days, her clinical condition deteriorated, with progression to continuous dystonia and chorea and hemiballism, that would interfere with daily activities. These symptoms worsened with stress and physical effort but disappeared during sleep. There were no facial signs, including angle of the mouth drooping, flattening of the nasolabial fold, or eyelid drooping. There was neither family history of a neurological disorder nor history of substance abuse, including cocaine and methamphetamine.

On examination, the patient was conscious with normal vital signs, well oriented in time and space, with hemodynamic and respiratory stability. Neurologically, she had a normal gait and standing posture. Muscle tone was normal, global and segmental muscle strength was retained, without any motor, sensory, or neurological deficits. The osteotendinous reflexes were symmetrical and of normal amplitude. The cranial nerves were intact. Babinski and cerebellar signs were absent. Systemic examination was unremarkable.

Laboratory results (Table 1) revealed hyperglycemia and elevated serum osmolality and cerebrospinal fluid (CSF) glucose levels but negative urinary ketones. Serology for hepatitis virus, HIV, and syphilis was negative. Hematological and endocrine assessments, including complete blood count with differentials and thyroid-stimulating hormone, were normal, as were serum ceruloplasmin levels and 24-hour urinary free copper levels. The diagnostic biomarkers of CSF 14-3-3 protein and tau for Creutzfeldt-Jakob disease were negative.

**Table 1:** Laboratory results on presentation. CSF: cerebrospinal fluid

Investigation	Result	Reference Range
Serum glucose, g/L	3.25	0.70–1.05
Hemoglobin A1c, %	13.5	3.8–6.5
CSF glucose, g/L	1.58	0.40–0.70
Blood osmolality, mOsm/kg	312.25	280–310
Sodium, mmol/L	139	137–147
Potassium, mmol/L	4.6	3.50–5.30

**Figure 1:**
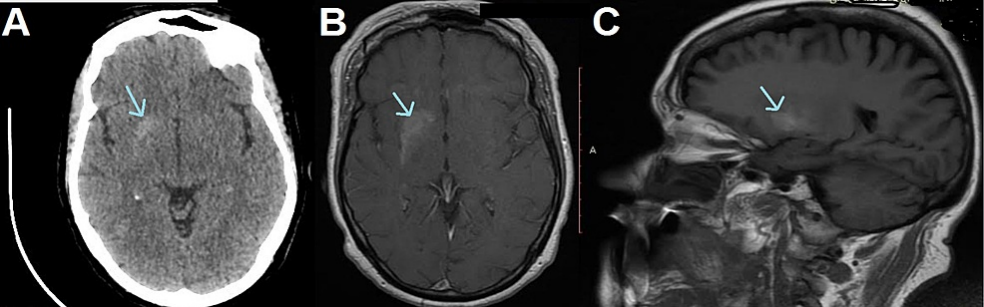
(A) Brain CT axial imaging showing hyperdensity of the right globus pallidum. (B, C) axial and sagittal MRI showing a hyperintense T1-weighted lesion in the striatal region.

A non-contrast CT head showed a hyperdense right globus pallidum. Brain MRI with gadolinium enhancement also revealed T1-weighted hyperintensity in the basal ganglia (Figure 1), consistent with a diagnosis of diabetic striatopathy.

It was decided to admit the patient and to start the patient on insulin. However, the patient refused admission and the team decided to prescribe oral antidiabetic medications since she was treatment-naive. She was prescribed metformin 1000 mg and glimepiride 4 mg, and was managed through telemedicine and outpatient visits. Oral administration of haloperidol 0.5 mg twice daily did not improve the neurological signs and symptoms. Subsequently, the dose was gradually increased to 1 mg twice a day. After three months of treatment, her hemichorea/hemiballismus significantly improved and subsequently resolved. Haloperidol was tapered and discontinued. As of this writing, there has been no relapse of dyskinesia. Diabetic counseling and medication compliance has been reinforced. Her hemoglobin A1c (HbA1c) improved from 13% to 7.5% over the three months period.

## Discussion

Non-ketotic hyperglycemia hemichorea/hemiballismus is a rare entity that occurs in the context of diabetes or acute hyperglycemia [7]. It is relatively more common in older female patients with type 2 diabetes [2].

Regarding the pathobiology of this syndrome, some possible mechanisms include: 1) hyperviscosity secondary to hyperglycemia leading to a regional disruption of the blood-brain barrier and metabolic failure [5]; 2) a lack of acetoacetate, due to a non-ketotic state, for gamma-aminobutyric acid (GABA) conversion and hence a decrease in GABA availability in the Striatum [5]; and 3) a hormonal theory, which postulates a heightened sensitivity of the post-menopausal dopamine receptor triggering hyperkinesis. This theory can possibly explain the strong predilection of this disease entity to present in women rather than men [5]. An autopsy study detected the presence of small lacunar infarcts with reactive astrocytosis and enhanced neuropeptide Y immunoreactivity in the putamen in these patients, similar to the pathologic changes in diabetic retinopathy [8, 9].

The diagnosis of diabetic striatopathy has three essential components. The first is clinical, that is, the chorea and hemiballism that are especially vigorous during a stressful or emotional state and disappear during sleep. Dystonia may also be present, as in our case. Previous studies have reported involvement of the tongue and face, but they were spared in our case. Dominant unilaterality of either the upper or lower limb is common [10]. In certain cases, this syndrome may herald a new diagnosis of diabetes [9, 11].

The second is evaluation of the serum glucose, HbA1c, and serum osmolality. An updated meta-analysis by Chua et al. [3] reported that at the time of diagnosis of diabetic striatopathy, the average serum glucose (414 mg/dL) and HbA1c (13.1%) is very high, consistent with the values found in our patient.

Imaging by MRI and/or CT is the third important element of diagnosis and complements the clinical examination; however, discrepancies in imaging depending on the modality have been reported. Nevertheless, MRI is more sensitive to striatal abnormalities than CT [3]; however, normal imaging does not rule out a diagnosis of diabetic striatopathy [9].

Typical CT head findings, at least with advanced disease, are hyperdensity of the striatal region, especially the caudate nucleus and putamen, similar to our case [10, 12]. Hyperintensity on T1 MRI with slight hypointensity in T2/fluid-attenuated inversion recovery (FLAIR) sequences is consistent with the diagnosis [13]. Imaging findings gradually disappear on resolution of hyperglycemia. However, normalization of the imaging may be slower than the clinical findings. Abnormal imaging can be seen for several months or even years after correction of the blood glucose [10, 14].

Therapeutic management includes strict glycemic control resulting in improvement of neurological symptoms. The symptoms may resolve over a longer period of a few months, as seen here. Anti-choreic drugs, like deutetrabenazine and tetrabenazine, may be indicated and can result in clinical improvement, especially if symptoms persist after correction of the hyperglycemia. Haloperidol has also been used and, if the patient does not respond, clonazepam, tetrabenazine, and tiapride may be considered [3]. The use of haloperidol depends on several factors; for example, advanced age and comorbidities may increase short-term mortality risk and hence the choice of the most suitable treatment [15]. In addition, surgical procedures, such as thalamotomy and deep brain stimulation, are also potential treatments in some rare refractory cases [16].

## Conclusions

Metabolic diabetic complications can be frequent and sometimes severe. Unfortunately, diabetic striatopathy is often misdiagnosed and remains an unfamiliar complication. The diagnostic work-up includes clinical assessment, glucose levels, and radiological examination. Early recognition and treatment are key to symptom and disease resolution.
